# Radiation Exposure during Cardiac Interventions in Congenital Heart Defects: A Multicenter German Registry Analysis 2012–2020

**DOI:** 10.1055/a-2514-7436

**Published:** 2025-03-04

**Authors:** Anja Tengler, Jörg Michel, Claudia Arenz, UIrike Bauer, Jens Beudt, Alexander Horke, Gunter Kerst, Andreas Beckmann, Michael Hofbeck

**Affiliations:** 1 Division of Pediatric Cardiology and Pediatric Intensive Care, Ludwig Maximilian University of Munich, Munich, Germany; 2 Department of Pediatric Cardiology, Pulmology and Pediatric Intensive Care, University Hospital Tuebingen, Tuebingen, Germany; 3 German Pediatric Heart Center, University of Bonn, Bonn, Germany; 4 National Register for Congenital Heart Defects, Berlin, Germany; 5 Division of surgery for Congenital Heart Defects, Department of Cardiac Surgery University of Hannover, Hannover, Germany; 6 Clinic for Pediatric Cardiology and Congenital Heart Disease, Klinikum Stuttgart, Stuttgart, Germany; 7 Clinic for Cardiac Surgery and Pediatric Cardiac Surgery, Heart Center Duisburg, Duisburg, Germany

**Keywords:** congenital heart disease, CHD, pediatric, cardiac catheterization/intervention (including PCI), stents, database

## Abstract

**Background** Interventional cardiac catheterizations have gained major importance in the treatment of congenital heart defects (CHDs). Since patients with CHDs frequently require lifelong medical care and sometimes subsequent invasive treatment, repeated radiation exposure during interventional procedures is a relevant issue concerning potential radiation-related risks. Therefore, a 9-year subanalysis on radiation data during interventional cardiac catheterizations from the German Registry for Cardiac Operations and Interventions in patients with CHDs was performed.

**Methods** The German Registry for Cardiac Operations and Interventions in Patients with CHDs is a real-world, prospective all-comers database collecting clinical and procedural data on invasive treatment of CHDs. From January 2012 until December 2020, a total of 28,374 cardiac catheter interventions were recorded. For a homogeneous case mix and for obtaining comparable data, eight specified interventions were selected for detailed evaluation. The selected procedures were: atrial septal defect (ASD)/patent foramen ovale (PFO) occlusion, patent ductus arteriosus (PDA) occlusion, ventricular septal defect (VSD) occlusion, coarctation of the aorta (CoA) balloon dilatation and stent implantation, aortic valvuloplasty, pulmonary valvuloplasty, and transcatheter pulmonary valve implantation (TPVI). Data on radiation exposure included total fluoroscopy time (TFT), dose area product (DAP), and DAP per body weight (DAP/BW).

**Results** The cohort accounted for 9,350 procedures, including 3,426 ASD/PFO occlusions, 2,039 PDA occlusions, 599 aortic and 1,536 pulmonary valvuloplasties, 383 balloon dilatations and 496 stent implantations for CoA, 168 VSD occlusions, and 703 TPVI. Six hundred and ten ASD/PFO procedures (17.8%) were performed without radiation. During the 9-year period, median annual TFT, DAP, and DAP/BW showed a continuous decrease while radiation burden correlated with intervention complexity: For ASD/PFO and PDA occlusion, aortic and pulmonary valvuloplasty as well as balloon dilatation of CoA the median DAP/BW was <20.0 μGy*m ^2^ /kg, while median values of 26.3 μGy*m ^2^ /kg and 31.6 μGy*m ^2^ /kg were noted for stent treatment of CoA and VSD closure, respectively. Radiation burden was highest in TPVI with a median TFT of 23.6 minutes, median DAP of 4,491 μGy*m ^2^ , and median DAP/BW of 79.4 μGy*m ^2^ /kg.

**Conclusion** A decrease in radiation exposure was found in eight cardiac interventions from January 2012 to December 2020. Comparison with international registries revealed a good quality of radiation protection. The data underline the requirement of surveillance of radiation burden, especially in this patient group.

## Introduction

Cardiac imaging based on ionizing radiation is essential for accurate diagnosis and treatment in patients with congenital heart defects (CHDs). 1 Based on significant technological improvements and innovations during the last decades, interventional transcatheter cardiac procedures have gained major importance in the treatment of congenital heart disease. 2 However, patients with complex CHDs represent a potentially vulnerable population since they frequently require repeat procedures during a lifelong treatment. 1 Despite constant improvements in cardiac catheterization equipment, the increasing number and the feasibility of performing sophisticated percutaneous interventions resulted in an expanding use of fluoroscopy and cineangiographies leading to a significant increase in radiation exposure. 3 4 Presently, in children with CHDs, diagnostic, interventional, and electrophysiological cardiac catheterizations account for more cumulative ionizing radiation than all other medical imaging modalities combined. 1 3 5 Since radiation exposure, especially in younger life, results in radiation-related risks including the potential risk of later development of cancer, 5 6 7 8 9 there is universal agreement that it should be kept as low as reasonably achievable without compromising diagnostic informative integrity and procedural safety. 1 10 Based on this knowledge, quality assurance and measures for radiation reduction have gained major importance for all professionals involved in the invasive treatment of patients with CHD. 1 7 10 11 12 In the past decades, several registries have been established to collect data on procedural risks and radiation exposure in congenital cardiac catheterization. 13 14 15 16 17 18 19 In 2012, the German Registry for Quality Assurance in CHD (Nationale Qualitätssicherung Angeborener Herzfehler) was founded by the German Society for Pediatric Cardiology and Congenital Heart Defects (DGPK) and the German Society for Thoracic and Cardiovascular Surgery (DGTHG). 20 21 This voluntary prospective, nationwide, multicenter registry collects treatment and outcome data of surgical and interventional procedures in patients suffering from CHD. 20 21 22 The purpose of this paper is to evaluate radiation exposure for pediatric cardiac interventions from January 2012 to december 2020 in this nationwide cohort and compare these data to those from international registries.

## Materials and Methods

The structure of the registry and data submission have been described in detail previously. 20 21 Inclusion criteria for the registry are the presence of any CHD and any invasive treatment by cardiac surgery or catheter-based intervention. Participation in this voluntary registry requires informed consent from either the patient or guardians, missing consent being the only exclusion criterion. The registry structure, data acquisition, and evaluation are in accordance with the guidelines of “Good Epidemiological Practice,” 23 “Good Clinical Practice,” 24 and the Declaration of Helsinki for medical research involving human subjects. 25 It was approved by Charité's Ethics Committee (code number: EA2/011/11).

Since the treatment of CHDs extends into adulthood, this patient group is also included. Each participating patient receives a unique personal identification (PID) for generating a pseudonym. Based on each PID, all cardiac surgical or interventional procedures performed in any participating German heart center can be assigned to an individual patient. The coding of diagnoses and procedures is based on the International Pediatric and Congenital Cardiac Code. 26 27 Prior to evaluation, the datasets are monitored with respect to data integrity and plausibility.

From January 2012 until December 2020, a total of 28,374 cardiac interventions were recorded in the German registry. To enable an analysis of homogenous patient groups and obtain comparable data for evaluation of radiation exposure, the following eight interventions were selected: Interventional occlusion of interatrial communications (secundum atrial septal defect [ASD] or patent foramen ovale [PFO]), patent ductus arteriosus (PDA) and ventricular septal defect (VSD), balloon valvuloplasty (BVP) of pulmonary valve stenosis (PSt) and aortic valve stenosis (AoSt), balloon dilatation and stenting of coarctation of the aorta (CoA), and transcatheter pulmonary valve implantation (TPVI; Table 1 ). Complete data on radiation were available for 9,350 procedures. According to the nature of the registry, these interventions included treatments of all age groups from neonates to adults. The dataset included patient characteristics, diagnoses, procedure type, total fluoroscopy time (TFT), and dose area product of radiation exposure (DAP). The DAP (surface integral of the air kerma) represents the product of radiation dose and the cross-sectional area exposed to the X-ray beam (μGy*m ^2^ ). 1 16 28 It summarizes both the sum of anteroposterior and lateral fluoroscopy and all cineangiographies. To provide an adjustment for the variability in dose among the wide range of patient age and weight in our cohort, we indexed DAP per body weight (DAP/BW) expressed as μGy*m ^2^ /kg. 1 16 17 18 28 Total air kerma (TAK) was not available for evaluation, since it was not part of the registry dataset during the observed period.

**Table 1:** Radiation exposure data (median, 75th, and 95th percentile) of eight selected interventional procedures

Procedure type	*N*	Median weight (5th–95th percentile, kg)	Median TFT (minutes)	75th	95th	Median DAP (μGy*m ^2^ )	75th	95th	Median DAP/BW (μGy*m ^2^ /kg)	75th	95th
ASD/PFO	2,816	44.6 (13–97)	5.0	9	18.5	213	648	2,900	5.8	13.6	48.2
ASD/PFO n.r.	610	33.9 (15–94)	–	–	–	–	–	–	–	–	–
PDA	2,039	13 (3.7–44.5)	7	11.1	21.4	103	214	846	8.4	16.9	48.1
AoSt	599	5 (2.6–61)	10	17	39.2	85	298	2,031	13.5	25.8	85.2
PSt	1,536	5.7 (2.4–68.5)	10.3	16	31.4	97	283	3,484	16.2	31.8	86.8
CoA BD	383	8.8 (3.1–77.8)	5.8	9.1	18.9	104	289	2,382	11	21.7	55.7
CoA Stent	496	51.6 (3.3–93.2)	9.3	13.4	23.4	1,178	2,854	8,434	26.3	52.8	117.9
VSD	168	22 (7.2–87.3)	18.7	31	48.2	695	1,702	12,524	31.6	57.8	227.4
TPVI	703	58.4 (23.3–99)	23.6	35	61	4,491	9,762	25,640	79.4	152.1	336.3

Abbreviations: AoSt, aortic valve stenosis; ASD/PFO, atrial septal defect/patent foramen ovale; ASD/PFO n.r., ASD/PFO occlusion without radiation; CoA, coarctation of the aorta; CoA BD, CoA treatment with balloon dilatation: CoA Stent, CoA treatment with stent implantation; DAP, dose area product; DAP/BW, dose area product per body weight; *N* , number of procedures; PSt, pulmonary valve stenosis; TFT, total fluoroscopy time; TPVI, transcatheter pulmonary valve implantation; VSD, ventricular septal defect.

Statistical analysis and charts were performed using SigmaPlot (Version 13 for Windows®, Systat Software Inc., San Jose, CA). Normality was assessed using the Shapiro–Wilk test. Data are presented as median and interquartile range (IQR). For further statistical analysis, the Student's *t* -test and the Mann–Whitney rank-sum test were applied, depending on the characteristics of data distribution. A probability value <0.05 was defined as statistically significant.

## Results

**Fig. 1:**
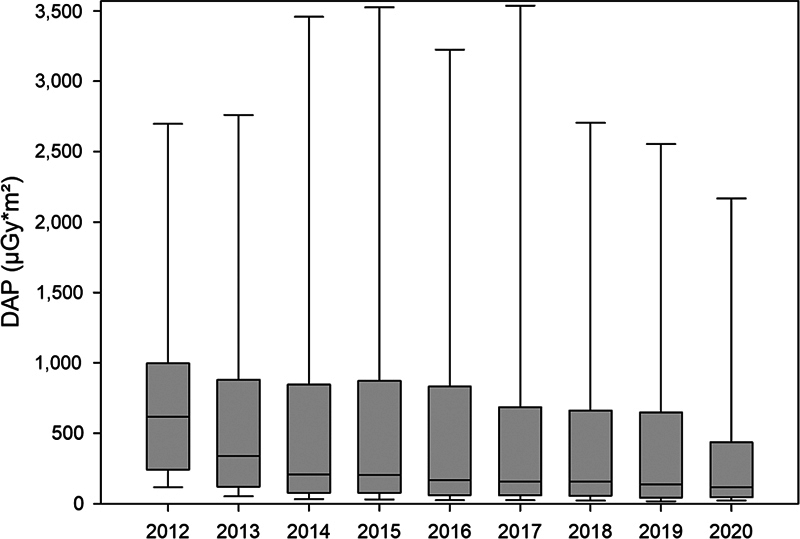
Box-and-whisker chart displaying median annual DAP (μGy*m ^2^ ) in the cohort of eight selected interventional procedures. The boxes represent the range of 25th to 75th percentile, the whiskers represent the statistically central range of data. DAP, dose area product.

**Fig. 2:**
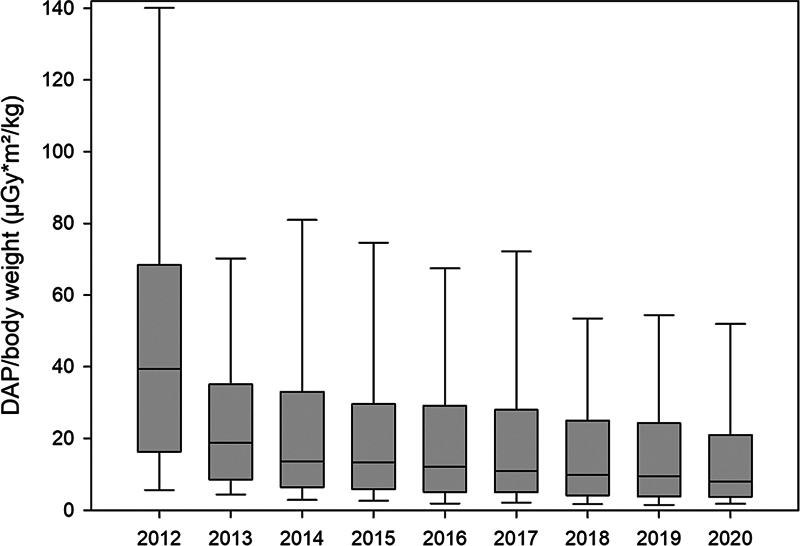
Box-and-whisker chart displaying median annual DAP/BW (μGy*m ^2^ /kg) in the cohort of eight selected interventional procedures. The boxes represent the range of 25th to 75th percentile, the whiskers represent the statistically central range of data. DAP/BW, dose area product per body weight.

During the 9-year period, the total number of interventions among the eight defined subgroups accounted for 9,350 procedures ( Table 1 ). Six hundred and ten ASD/PFO occlusions (6.5% of the entire 9,350 procedures) were performed without radiation. A comparison of the median annual fluoroscopy time (TFT) of the remaining 8,740 procedures performed with radiation revealed a major decrease from 2012 to 2014, followed by a further small decrease from 2015 to 2020 ( Table 2 ). Equal trends were observed in the median annual DAP and DAP/BW ( Table 2 and Figs. 1 and 2 ).

**Table 2:** Annual radiation exposure data from 2012 to 2020 (median, 75th, and 95th percentile) of eight selected interventional procedures

Year	*N*	Median TFT (minutes)	75th	95th	Median DAP (μGy*m ^2^ )	75th	95th	Median DAP/BW (μGy*m ^2^ /kg)	75th	95th
2012	26	13.8	25.7	41.2	619	998	2,951	39.5	68.4	214.7
2013	273	10	17.8	37.7	338	881	6,333	18.8	35.1	100.9
2014	1,153	8	13.4	34.8	209	845	6,717	13.6	33	133.1
2015	1,191	8.3	14	32	203	873	6,683	13.4	29.7	114.5
2016	1,309	8	14	32.3	169	832	5,765	12.2	29.1	102.2
2017	1,292	8.2	15	34.5	158	686	6,466	10.9	28	110.6
2018	1,214	8	13.5	32.1	159	661	5,104	9.9	25	78.8
2019	1,125	7.1	13	31.8	135	649	5,251	9.5	24.3	94.2
2020	1,157	7.1	12.2	31	117	436	4,940	8	21	87

Abbreviations: DAP, dose area product; DAP/BW, dose area product per body weight; *N* , number of procedures; TFT, total fluoroscopy time.

**Fig. 3:**
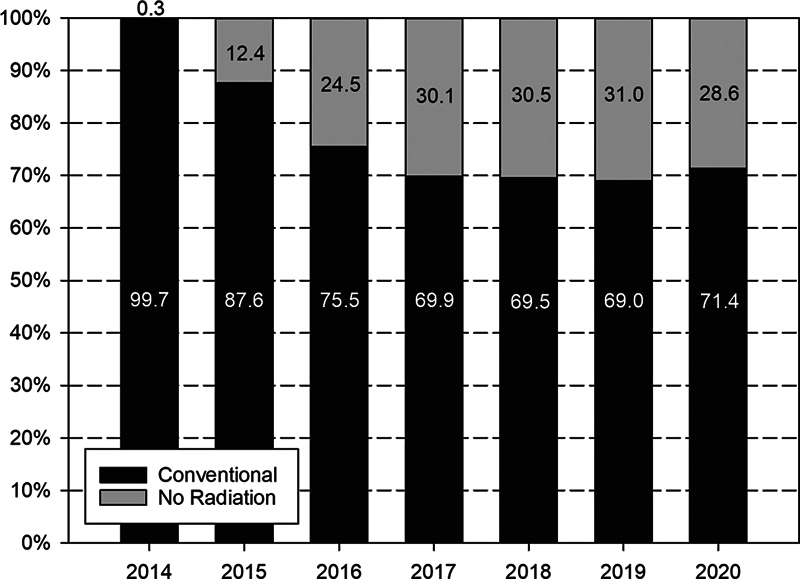
Annual percentages of interventional occlusion of ASD/PFO with (black columns) and without (gray columns) radiation in the German registry. ASD/PFO, atrial septal defect/patent foramen ovale.

Among the eight selected interventional procedures, the most frequent one was the closure of secundum ASD or PFO (3,426 procedures). Interventional occlusion of PFO accounted for 887 (25.9%%). Six hundred and ten interventions (17.8%) for closure of ASD or PFO were performed without radiation under echocardiographic guidance ( Table 1 ). This trend started in 2014, resulting in approximately 30% radiation-free procedures for ASD/PFO occlusion during the last 4 study years ( Fig. 3 ). Median TFT of the 2,816 procedures under fluoroscopic guidance was 5 minutes, median DAP was 213 μGy*m ^2^ , and median DAP/BW 5.8 μGy*m ^2^ /kg ( Table 1 ).

The second most frequent procedure ( *n* = 2,039) was PDA occlusion with a median TFT of 7 minutes, a median DAP of 103 μGy*m ^2^ , and a median DAP/BW of 8.4 μGy*m ^2^ /kg ( Table 1 ). Specification of the implanted device was available for 1,418 procedures: Occlusion was achieved by a duct-occluder in 636 and by coils in 782 interventions. Median TFT was significantly longer in procedures requiring implantation of duct-occluders (7.3 vs. 6 minutes, *p* < 0.001) compared with procedures performed with coil occlusion ( Table 3 ). Patients who received duct-occluders were smaller (median weight 10.25 kg vs. 16.6 kg, *p* < 0.001), probably presenting with larger PDAs at a younger age. A mild but statistically significant difference (8.0 vs. 7.2 μGy*m ^2^ /kg, *p* = 0.03) was observed regarding median DAP/BW ( Table 3 ).

**Table 3:** Radiation exposure during different techniques of interventional patent ductus arteriosus occlusion

Procedure type	*N*	Median weight, kg	Median TFT (minutes)	Median DAP (μGy*m ^2^ )	Median DAP/BW (μGy*m ^2^ /kg)
PDA-DO	636	10.25	7.3	82	8
PDA-Coil	782	16.6	6	112	7.2
PDA all procedures	2,039	13	7	103	8.4

Abbreviations: DAP, dose area product; DAP/BW, dose area product per body weight; *N* , number of procedures; PDA, patent ductus arteriosus; PDA-Coil, PDA-occlusion by coil implantation; PDA-DO, PDA occlusion by implantation of a duct-occluder; TFT, total fluoroscopy time.

**Fig. 4:**
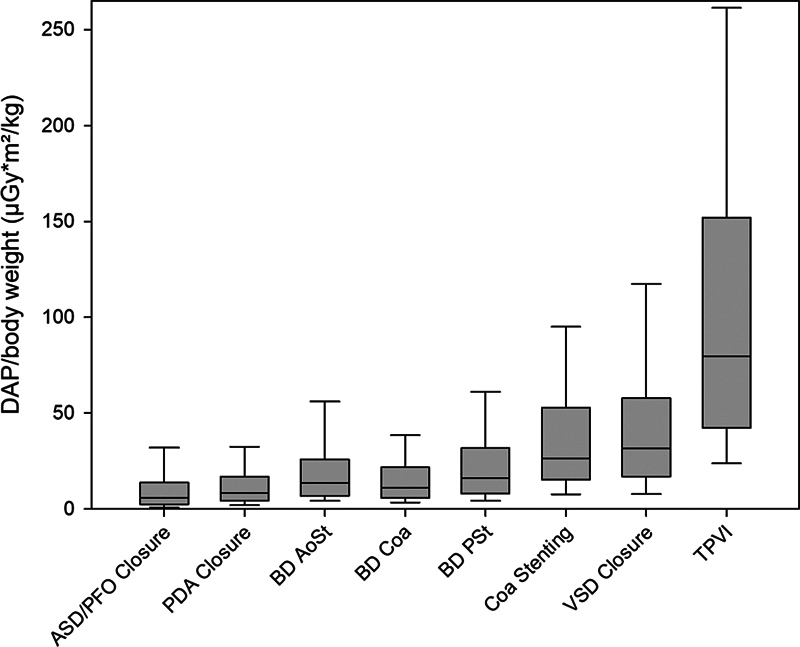
Box-and-whisker chart displaying median DAP/BW (μGy*m ^2^ /kg) among the eight selected interventional procedures. The boxes represent the range of 25th to 75th percentile, the whiskers represent the statistically central range of data. AoSt, aortic valve stenosis; ASD/PFO, atrial septal defect/patent foramen ovale; BD, balloon dilatation; CoA, coarctation of the aorta; DAP/BW, dose area product per body weight; PDA, patent ductus arteriosus; PSt, pulmonary valve stenosis; TPVI, transcatheter pulmonary valve implantation; VSD, ventricular septal defect.

Median TFT, DAP, and DAP/BW showed similar results in the subgroups of pulmonary (1,536 procedures) and aortic (599 procedures) balloon valvuloplasty (BVP; Table 1 and Fig. 4 ). CoA (879 procedures) was treated either by angioplasty (383 procedures) or stent implantation (496 procedures; Table 4 ). While balloon dilatation was performed more frequently in smaller patients (median weight 8.8 kg), stent implantation was more common among larger patients (median weight 51.6 kg, *p* < 0.001). Balloon angioplasty (BAP) of CoA was associated with significantly lower values of median TFT, median DAP, and median DAP/BW ( *p* < 0.001; Table 4 ).

**Table 4:** Radiation exposure during different techniques of coarctation of the aorta treatment

Procedure type	*N*	Median weight, kg	Median TFT (minutes)	Median DAP (μGy*m ^2^ )	Median DAP/BW (μGy*m ^2^ /kg)
CoA-BD	383	8.8	5.8	104	11
CoA-Stent	496	51.6	9.3	1,178	26.3
CoA all procedures	879	25	7.9	369	19

Abbreviations: CoA, coarctation of the aorta; CoA-BD, balloon dilatation of CoA; CoA-Stent, CoA treatment by stent implantation; DAP, dose area product; DAP/BW, dose area product per body weight; *N* , number of procedures; TFT, total fluoroscopy time.

One hundred and sixty-eight procedures were performed for interventional VSD occlusion. The complexity of these procedures resulted in a median TFT of 18.7 minutes ( Table 1 ). Median DAP (695 μGy*m ^2^ ) and median DAP/BW (31.6 μGy*m ^2^ /kg) were higher compared with interventional ASD/PFO occlusion, BVP of aortic and pulmonary stenosis and treatment of CoA ( Fig. 4 ). Among all procedures, TPVI (703 procedures) required the longest median TFT (23.6 minutes) as well as the highest median DAP (4,491 μGy*m ^2^ ) and median DAP/BW (79.4 μGy*m ^2^ /kg; Table 1 and Fig. 4 ).

## Discussion

This is the first analysis from the German Registry in CHDs focusing on radiation exposure data during interventions from January 2012 to December 2020. Since 2012, the German Registry for Cardiac Operations and Interventions in CHD has collected specific data on surgical and interventional treatment of CHDs. Registry data are published annually containing detailed information on the entire cohort, various subgroups, and 15 index procedures ( www.dgpk.org , www.dgthg.org ). In addition, each participating institution receives annual benchmark reports comparing the institutional data to the nationwide results.

There is a worldwide consensus that TFT alone is not a sufficient parameter for the description of radiation exposure, since it does not take into account patient size, beam angulation, use of cineangiography, and other relevant factors. 7 Nevertheless, TFT provides hints regarding the complexity and duration of the procedure. A TFT of >60 minutes has been defined as a substantial value that should trigger follow-up for early detection and management of possible skin injuries. 7

TAK could not be included in our evaluation since this parameter was introduced into the dataset of this registry only in 2022. It represents the X-ray energy delivered to the air at a defined distance from the X-ray tube focal spot. 1 7 TAK is used as a predictor of the risk of threshold-dependent deterministic skin effects. 1 7 Since the likelihood of skin reaction is greater in adults, for whom a higher radiation dose is required to achieve adequate image quality, TAK is a less important parameter in children. 1 DAP is generally considered the most important parameter for the description of radiation exposure since it represents a surrogate of stochastic, non-threshold cancer risk to the patient. 1 7 DAP is the cumulative sum of the product of instantaneous air kerma and X-ray field area (air kerma − area product), reflecting the total radiation emitted by the X-ray tube. 1 7 DAP/BW (μGy*m ^2^ /kg) has been introduced as a surrogate for the delivered energy indexed to body weight. 12 28 This parameter has not been validated as a method of radiation exposure yet. However, it seems to be valuable for allowing comparison of patient groups comprising a wide range of weight and age. 11 12 16 18 28 29

During the study period, the median fluoroscopy time of the eight interventional procedures in our cohort showed a major decrease during the first 3 years, followed by some further mild decrease during the remaining years ( Table 2 ). Median annual DAP and median DAP/BW showed similar trends, resulting in a median DAP <200 μGy*m ^2^ and a median DAP/BW <10 μGy*m ^2^ /kg in the past 3 years ( Table 2 and Figs. 1 and 2 ). Similar trends of reduced radiation burden over time were recorded by Harbron et al, who showed a significant decrease in median DAP during cardiac catheterizations in children and young adults in three United Kingdom hospitals from 1994 until 2013. 11 This trend was also evident among six selected interventional procedures including ASD and PDA occlusion, pulmonary and aortic valvuloplasty, and pulmonary artery and CoA angioplasty. 11

In accordance with other registries, the present study focused on the evaluation of the most frequent, well-defined procedures to obtain comparable data. 12 14 28 These procedures included occlusion of ASD/PFO, PDA and VSD, pulmonary and aortic valve valvuloplasty, and treatment of CoA and TPVI ( Tables 1 and 5 ). Data on fluoroscopy requirements during these interventions correlated with their type and complexity. Interventional closure of ASD/PFO, PDA, and BAP of CoA required relatively short median fluoroscopy times of 5 to 7 minutes. BVP of the aortic and pulmonary valve as well as stenting of CoA required a median TFT of 9 to 11 minutes. Significantly longer fluoroscopy times were noted for VSD occlusion (median TFT 18.7 minutes) reflecting the requirement of fluoroscopy during the complex steps of this intervention. 30 In accordance with other registries, the longest median TFT (23.6 minutes) was required in TPVI ( Tables 1 and 5 ). 12 14 28 29

**Table 5:** Comparison of radiation exposure data in the literature

Procedure type	Study	Study period	N	TFT	Median DAP	Median DAP/BW
ASD	Ghelani et al 14	January 2009–July 2013	731	18	2,100	–
	Cevallos et al 28	January 2014–June 2015	307	17	771	34
	Lamers et al 12	February 2014–August 2016	58	12	229	9
	Sitefane et al 36	January 2009–November 2015	174	1	88	3.2
	GR	January 2012–December 2020	2,816	5	213	5.8
PDA	Ghelani et al 14	January 2009–July 2013	548	12	700	–
	Cevallos et al 28	January 2014–June 2015	463	13	407	37
	Lamers et al 12	February 2014–August 2016	109	13	135	15
	GR	January 2012–December 2020	2,039	7	103	8.4
BD AoSt	Ghelani et al 14	January 2009–July 2013	297	25	1,400	–
	Cevallos et al 28	January 2014–June 2015	140	27	959	99
	Lamers et al 12	February 2014–August 2016	25	15	394	34
	GR	January 2012–December 2020	599	10	85	13.5
BD PSt	Ghelani et al 14	January 2009–July 2013	462	20	700	–
	Cevallos et al 28	January 2014–June 2015	267	18	326	53
	Lamers et al 12	February 2014–August 2016	45	16	116	21
	GR	January 2012–December 2020	1,536	10.3	97	16.2
CoA	Ghelani et al 14	January 2009–July 2013	452	22	2,900	–
	Cevallos et al 28	January 2014–June 2015	299	23	1,307	90
	Lamers et al 12	February 2014–August 2016	46	19	598	45
	GR	January 2012–December 2020	879	7.9	369	19
TPVI	Ghelani et al 14	January 2009–July 2013	223	55	23,000	–
	Cevallos et al 28	January 2014–June 2015	204	43	13,551	257
	Lamers et al 12	February 2014–August 2016	17	51	9,869	197
	Goldstein et al 29	January 2014–December 2016	530	42	10,169	198
	GR	January 2012–December 2020	703	23.6	4,491	79.4

Abbreviations: AoSt, aortic valve stenosis; ASD, atrial septal defect; BD, balloon dilatation; CoA, coarctation of the aorta; DAP, dose area product; DAP/BW, dose area product per body weight; GR, German registry; N, number of procedures; PDA, patent ductus arteriosus; PSt, pulmonary valve stenosis; TFT, total fluoroscopy time; TPVI, transcatheter pulmonary valve implantation.

Median DAP is given as μGy*m ^2^ , median DAP/BW as μGy*m ^2^ /kg.

Similar to TFT, median values of DAP and DAP/BW correlated with the complexity and nature of the underlying procedures ( Table 1 and Fig. 4 ). ASD occlusion, the most frequent interventional procedure, had a median DAP of 213 and DAP/BW of 5.83 μGy*m ^2^ /kg, which are significantly lower than data reported in most of the previous studies ( Table 5 ). 12 14 28 For this comparison, it has to be taken into account that 25.9% of the investigated German cohort were PFO occlusions (887/3,426). This information is not available from other registries. An important trend in the interventional treatment of secundum ASD and PFO in the German registry has been the fact that a significant proportion of patients was treated without radiation ( Fig. 3 ). This alternative approach, using guidance during the procedure by transesophageal echocardiography, has been described as safe and effective. 31 During the past 4 years, approximately 30% of the procedures of secundum ASD and PFO occlusion were performed without radiation ( Fig. 3 ). To the best of our knowledge, this trend has not been reported in other registries so far.

BVP of pulmonary and aortic stenosis as well as PDA occlusion was performed with a median DAP below or slightly above 100 μGy*m ^2^ and DAP/BW <20 μGy*m ^2^ /kg. While higher radiation doses were required in patients with treatment of CoA, these doses still compare favorably with data from other registries ( Tables 4 and 5 ). 12 14 28 Patients undergoing interventional treatment of CoA showed differences according to the treatment mode. Balloon dilatation resulted in significantly less radiation burden than treatment requiring stent implantation ( Table 4 ). This can be explained by the lower weight of children treated by BAP and the requirement for more angiographies in those treated by stent implantation. There are only few data available in the literature regarding radiation exposure during interventional VSD occlusion. Quinn et al classified VSD device closure with additional intervention among procedures of the medium exposure category. 16 The medium DAP/BW in VSD occlusions was 108 μGy*m ^2^ /kg as compared with the medium value in our cohort of 31.6 μGy*m ^2^ /kg. According to data from the C3PO-QI project, the seven procedures in the German registry mentioned so far could be classified in the low radiation exposure category defined as median DAP/BW <100 μGy*m ^2^ /kg. 16

As expected, TPVI was associated with the highest median values of DAP and DAP/BW ( Table 1 and Fig. 4 ). Nevertheless, median DAP (4,491 μGy*m ^2^ ) and DAP/BW (79.4 μGy*m ^2^ /kg) compare very favorably to previously published data ( Table 5 ). 12 28 29 Some differences between our data and other registries may be explained by the use of different devices and by the fact that some centers prefer to perform balloon testing of the coronary arteries, stenting of the right ventricular outflow tract, and pulmonary valve implantation as a single-step procedure while others perform testing and preparation of the right ventricular outflow tract, followed by prosthetic valve implantation, as separate procedures. 32 33 34 Future devices may be associated with a different radiation burden. 35 Especially in this population with a dysfunctional right ventricular outflow tract, careful registration of radiation data is necessary, since these patients frequently require repeat cardiac catheterizations during their lifelong treatment.

The benefit of structured quality improvement measures for the reduction of radiation exposure was demonstrated by Cevallos et al. 28 Comparison of procedure-specific radiation dose data among institutions participating in the C3PO-QI project from January 2009 to July 2013 to data from January 2014 to June 2015 showed a significant decrease of all radiation parameters. 14 28 Substantial improvement in radiation exposure was also recorded in a prospective study performed by Quinn et al from January 2015 to December 2017. 17 Based on the implementation of targeted interventions, addressing selected strategic domains for radiation reduction, median DAP decreased by 30% for all procedures in this cohort. 17 Remarkably low-radiation dose parameters during interventional occlusion of ASD were achieved by Sitefane et al based on specific measures to reduce radiation exposure during this type of intervention ( Table 5 ). 36 Significant reduction of radiation burden in patients with CHDs can also be achieved by improvement of radiology equipment or changes in examination techniques. 12 37

The continuous improvement of radiation exposure observed in this registry over the 9-year period cannot be attributed to a specific program. It appears to be the result of a general awareness, the introduction of noninvasive imaging in the preparation of the procedures, and continuous improvement of the technical equipment. The significant percentage of ASD and PFO occlusions performed without radiation underlines the ambition of the centers involved to reduce radiation exposure as much as possible ( Fig. 3 ). Establishment of robust benchmark databased on large cohorts of well-defined procedures will be extremely important for future improvements in this field. In this respect, further valuable contributions could be derived from the dataset of the German Registry. Comparison to other registries shows that interventional treatment of CHDs in Germany is offered with high quality concerning radiation exposure of treated patients.

## Limitations

The registry is limited by the voluntary participation of patients and institutions even though its principle is an all-comers design. The dataset of the German Registry does not include information about the infrastructure and equipment of catheterization laboratories. During the first 2 years of the registry data on radiation exposure were designated as optional fields in the dataset. Therefore, the potential impact of the smaller number of patients included from 2012 to 2013 cannot be excluded. Since this is the first report on radiation exposure data of the registry, evaluation required substantial time, resulting in some delay in the preparation of this manuscript.

## Collaborators

### German Quality Assurance/Competence Network for Congenital Heart Defects

Ulrike Herberg, Majed Kanaan, Corinna Lebherz, Stefan Ostermayer (Aachen); Stephan Schubert, Kai Thorsten Laser (Bad Oeynhausen); Felix Berger, Oliver Miera, Bernd Opgen-Rhein, (Berlin); Johannes Breuer, Martin Schneider, Nicole Müller (Bonn); Trong Phi Lê, Konstantin Papakostas (Bremen); Gleb Tarusinov, Aktham Tannous, Paul Hacke (Duisburg); Ertan Mayatepek (Düsseldorf); Sven Dittrich, Wolfgang Wällisch (Erlangen); Carsten Müntjes (Essen); Brigitte Stiller, Alexej Bobrowski, Charlotte Schwab, Christoph Zürn, Daniela Kocher, Hannah Kappler, Hendryk Schneider, Lisa Marie Stelling - Fuchs, Meike Schwendt, Miriam Schwab, Simon Oberle, Thilo Fleck (Freiburg); Christian Jux (Gießen); Thomas Paul, Matthias Sigler, Heike Schneider, Matthias Müller, Ulrich Johannes Krause (Göttingen); Roland Haase (Halle), Ulrike Issa, Caroline Schmitt; Rainer Kozlik-Feldmann, Carsten Rickers, Lena Christine Siebel, Philipp Schneider, Roland Volker Jebens, Veronika Stark (Hamburg); Philipp Beerbaum, Dietmar Böthig (Hannover); Matthias Gorenflo, Sebastian Uhl (Heidelberg); Hashim Abdul-Khaliq, Axel Rentzsch (Homburg); Thomas Kriebel, Michael Zimmer, Peter Follmann (Kaiserslautern); Anselm Uebing, Gunther Fischer, Jan-Hinnerk Hansen Kolja Becker, Ulrike Hoffmann (Kiel); Markus Khalil (Köln), Ursula Bangen, Verena Strunz; Ingo Dähnert, Frank-Thomas Riede (Leipzig); Christoph Kampmann, Tariq Abu-Tair (Mainz); Peter Ewert, Kilian Ackermann, Gunter Balling, Kristina Borgmann, Andreas Eicken, Julia Elmenhorst, Thomas Genz, Christoph Röhlig (München); Nikolaus A. Haas, André Jakob, Christoph Funk, Felix Sebastian Oberhoffer, Guido Mandilaras, Matthias Hermann (Großhadern, München); Hans-Gerd Kehl, Felix Kleinerüschkamp, Volker Debus, Helmut Baumgartner (Münster); Matthias W. Freund, Gerrit Kopiske, Michael Schumacher, (Oldenburg); Gunther Kerst, Ulrich Schweigmann, Volker Ocker (Stuttgart); Johannes Nordmeyer, Jörg Michel, Christan Scheckenbach, Vanya Icheva (Tübingen); Christian Apitz, Michael Kästner (Ulm); Kai O. Hensel, Andreas Heusch (Wuppertal).

### Participating Centers Entering Data on Cardiac Interventions from 2012 to 2020

Universitätsklinikum Aachen; Kinderherzzentrum/Zentrum für angeborene Herzfehler Bad Oeynhausen; Deutsches Herzzentrum der Charité, Berlin; Universitätsklinikum Bonn; Kinderkardiologie Bremen; Herzzentrum Duisburg; Kinderkardiologie Düsseldorf Universitätsklinikum Erlangen; Kinderkardiologie Essen; Universitäts-Herzzentrum Freiburg/Bad Krozingen; Universitätsklinikum Gießen; Universitätsklinikum Göttingen; Universitätsklinikum Halle; Universitätsklinikum Hamburg-Eppendorf; Medizinische Hochschule Hannover; Universitätsklinikum Heidelberg; Universitätsklinikum des Saarlandes, Homburg; Westpfalz-Klinikum Kaiserslautern; Universitätsklinikum Schleswig-Holstein, Kiel; Universitätsklinikum Köln; Herzzentrum Leipzig; Universitätsklinikum Mainz; DHZ München; Klinikum der LMU Campus Großhadern, München; Universitätsklinikum Münster; Klinikum Oldenburg; Klinikum Stuttgart; Universitätsklinikum Tübingen; Universitätsklinikum Ulm; Universitätsklinikum Wuppertal
